# Thermal Properties of SiOC Glasses and Glass Ceramics at Elevated Temperatures

**DOI:** 10.3390/ma11020279

**Published:** 2018-02-10

**Authors:** Christina Stabler, Andreas Reitz, Peter Stein, Barbara Albert, Ralf Riedel, Emanuel Ionescu

**Affiliations:** 1 Institut für Materialwissenschaft, Technische Universität Darmstadt, Otto-Berndt-Straße 3, D-64287 Darmstadt, Germany; stabler@materials.tu-darmstadt.de (C.S.); p.stein@mfm.tu-darmstadt.de (P.S.); riedel@materials.tu-darmstadt.de (R.R.); 2 Eduard-Zintl-Institut für Anorganische und Physikalische Chemie, Technische Universität Darmstadt, Alarich-Weiss-Straße 12, D-64287 Darmstadt, Germany; reitz@ac.chemie.tu-darmstadt.de (A.R.); albert@ac.chemie.tu-darmstadt.de (B.A.)

**Keywords:** silicon oxycarbide, silicon oxide carbide, thermal transport, thermal conductivity, thermal expansion

## Abstract

In the present study, the effect of the chemical and phase composition on the thermal properties of silicon oxide carbides (SiOC) has been investigated. Dense monolithic SiOC materials with various carbon contents were prepared and characterized with respect to their thermal expansion, as well as thermal conductivity. SiOC glass has been shown to exhibit low thermal expansion (e.g., ca. 3.2 × 10^−6^ K^−1^ for a SiOC sample free of segregated carbon) and thermal conductivity (ca. 1.5 W/(m∙K)). Furthermore, it has been observed that the phase separation, which typically occurs in SiOC exposed to temperatures beyond 1000–1200 °C, leads to a decrease of the thermal expansion (i.e., to 1.83 × 10^−6^ K^−1^ for the sample above); whereas the thermal conductivity increases upon phase separation (i.e., to ca. 1.7 W/(m∙K) for the sample mentioned above). Upon adjusting the amount of segregated carbon content in SiOC, its thermal expansion can be tuned; thus, SiOC glass ceramics with carbon contents larger than 10–15 vol % exhibit similar coefficients of thermal expansion to that of the SiOC glass. Increasing the carbon and SiC content in the studied SiOC glass ceramics leads to an increase in their thermal conductivity: SiOC with relatively large carbon and silicon carbides (SiC) volume fractions (i.e., 12–15 and 20–30 vol %, respectively) were shown to possess thermal conductivities in the range from 1.8 to 2.7 W/(m∙K).

## 1. Introduction

Silicon oxide carbide (or silicon oxycarbide, SiOC) glasses and glass ceramics belong to the group of polymer-derived ceramics (PDCs). They consist of corner-sharing SiO_4−x_C_x_ tetrahedra (x = 0–4) [1], and can be described as vitreous silica glasses, with oxygen being partly replaced by carbon within the glass network. The main synthesis approach, which incorporates significant amounts of carbon into silica, relies on the thermal conversion of sol–gel derived precursors based on organo-substituted alkoxysilanes or of polyorganosiloxanes [2]. A significant amount of Si–O and Si–C bonds were preserved upon the thermal treatment, yielding X-ray amorphous SiOC at 1000 °C. The carbon was present as network carbon, i.e., bonded to silicon (sp^3^-hybridized; SiO_4−x_C_x_ tetrahedra) as well as in form of disordered segregated carbon phase (sp^2^-hybridized) [3] homogeneously dispersed inside the glassy matrix. The final composition of the SiOC glasses can be tuned upon choosing appropriate preceramic precursors [2,4,5].

Silicon oxide carbide glasses were shown in various studies to undergo phase separation at temperatures beyond 1200 °C. As evidenced by solid-state Magic Angle Spinning ^29^Si NMR (MAS NMR) measurements, the glassy matrix of SiOC glasses is continuously evolving in the temperature range between 1200–1600 °C [6]. At temperatures between 1000–1200 °C, the SiOC glass matrix consists of SiO_4−x_C_x_ (x = 0 to 4) mixed-bonds silicon tetrahedra [1]. These materials are fully X-ray amorphous [7,8], and can consequently be addressed as SiOC glasses. At temperatures exceeding 1200 °C, the phase separation of the glassy matrix starts, and the mixed bonds containing SiO_3_C, SiO_2_C_2_, and SiOC_3_ tetrahedra disappear [6]. Thus, in SiOC samples exposed to high temperatures, e.g., 1600 °C, no mixed-bonds tetrahedra are detectable by ^29^Si MAS NMR spectroscopy. At the same time, β-SiC nanoparticles become visible in XRD upon phase separation and crystallization [9]. However, there are no indications of the crystallization of cristobalite up to temperatures of even beyond 1500 °C [10]. Consequently, these samples can be regarded as glass ceramics consisting of a silica matrix in which homogeneously dispersed nanoparticles of β-SiC and a sp^2^-hybridized segregated carbon phase are present.

The concept of phase separation is especially important, as for instance SiOC materials prepared at around 1000 °C will be different if they are exposed to working temperatures exceeding 1200 °C. Therefore, it is necessary to know the thermal properties of both SiOC glasses and glass ceramics. Moreover, the chemical composition is known to govern various properties of SiOC materials, e.g., oxidation [11,12] and crystallization resistance [8]. Consequently, for an optimal design of SiOC suitable for applications at high temperatures and in harsh environments, the impact of the chemical composition on the thermal properties must be known.

SiOC glasses exhibit high stability with respect to decomposition and crystallization up to temperatures beyond 1000 °C, and furthermore show excellent behavior in oxidative and corrosive environments [2]. Thus, SiOC glasses are potential high-temperature materials for application in internal combustion engines, high-temperature reactors, heat exchangers, etc. For such applications, knowledge of the thermal properties of SiOC glasses and glass ceramics is of great interest.

Several studies report on the thermal expansion of SiOC-based materials. Typically, values for the coefficient of thermal expansion (hereafter CTE) are reported to be close to 3 ppm/K for pure SiOC systems [13,14,15]. Some studies show the possibility of tailoring the CTE of SiOC-based materials. The CTE can be increased up to 6.6 ppm/K upon the incorporation of additional phases, either by the pyrolysis of metal-modified preceramic precursors yielding Si(M)OC nanocomposites [16], or by the use of highly conductive fillers [15]. In a similar manner, the CTE can as well be reduced. Very recently, Fedorova et al. reported on the synthesis of SiOC materials containing β-eucryptite that exhibit a near-zero CTE [17].

Less information is available in the literature on the thermal transport of SiOC materials. Mazo et al. and Eom et al. reported thermal conductivities of silicon oxide carbides ranging from ca. 1.3 to ca. 1.8 W/(m∙K) at room temperature [18,19,20]. Gurlo et al. measured the temperature-dependent thermal conductivity of a SiOC glass and glass ceramic with the same chemical composition [21]. The SiOC glass showed a very low thermal conductivity of about 0.5 W/(m∙K). However, the investigated monolith exhibited a significant porosity of ca. 12 vol %. Consequently, in the mentioned study, no clear distinction between the impact of phase separation and porosity on the thermal properties of silicon oxide carbide was possible. Upon increasing the porosity in SiOC to approximately 80 vol %, the thermal conductivity can be decreased to values of about 0.041–0.078 W/(m∙K) [22]. Despite the mentioned studies in the literature reporting on the low intrinsic thermal conductivity of SiOC-based materials, there is no systematic assessment of the thermal transport in silicon oxide carbides available, which tries to rationalize, for instance, the effect of the composition and nano/microstructure of SiOC on its thermal conductivity. Thus, in the present study, we evaluate the effect of the composition of SiOC on its thermal properties, i.e., thermal expansion as well as thermal transport. Moreover, we address the aspect of whether, and to which extent, the phase separation that occurs at high temperatures in silicon oxide carbide glasses affects their thermal conductivity.

## 2. Experimental Procedure

*Materials Synthesis.* SiOC glasses and glass ceramic samples with four different compositions, mainly with varying contents of segregated carbon (C1–SiOC, C12–SiOC, C16–SiOC, and C17–SiOC; C1, C12, C16, and C17 indicate the approximate volume fraction of segregated carbon in the prepared samples), were synthesized for this study. Monolithic specimens were obtained by uniaxial hot pressing silicon oxide carbide powders at 1600 °C (monolithic samples were denoted as C1–SiOC–1600, C12–SiOC–1600, C16–SiOC–1600, and C17–SiOC–1600). Additionally, monolithic samples of C1–SiOC were prepared in a pressureless pyrolysis process at 1100 °C (monolithic sample denoted as C1–SiOC–1100). Suitable monolithic coupons were cut from the obtained monolithic samples with a diamond wire cutter, and used for the experiments.

C12–SiOC and C17–SiOC were prepared from the commercially available polymethylsilsesquioxane Belsil PMS MK (Wacker GmbH, Burghausen, Germany), and polysiloxane SPR–212 (Starfire Systems Inc., NY, USA), respectively. Both polymers were cross-linked in an alumina tube furnace at 250 °C for two hours under flowing argon atmosphere. Subsequently, the cross-linked samples were heated at 1000 °C, with a holding time of two hours. The heating and cooling rates were 100 °C/h. The obtained glassy chunks were crushed and sieved to a grain size of < 40 µm. Hot pressing of C12–SiOC was performed at 1600 °C in a cylindrical graphite die at a constant pressure of 50 MPa in static argon atmosphere. The temperature was held at 1600 °C for 30 min before cooling down. C17–SiOC was densified upon spark plasma sintering (SPS) at 1600 °C for 15 min in a cylindrical graphite die.

C16–SiOC was prepared according to Radovanovic et al. [23] upon the sol–gel processing using 80 wt % polymethylhydrosiloxane (PMHS; average M_n_ 1700–3200; Merck, Germany) and 20 wt % 1,3,5,7-tetramethyl-1,3,5,7-tetravinylcyclotetrasiloxane (D_4_Vi; 97%, ABCr, Karlsruhe, Germany). After 30 min stirring at 0 °C, 1 wt % platinum catalyst (Platinum(0)-1,3-divinyl-1,1,3,3-tetramethyl-disiloxane complex solution; 0.1 M in poly(dimethylsiloxane) vinyl terminated; Merck, Germany) was added and homogenized for five minutes. The mixture was subsequently filled in polystyrene boxes with a closed lid. After 30 min at 0 °C, the samples were stored for 24 h at −15 °C. The xerogels obtained were pyrolyzed at 1000 °C, crushed, sieved to < 40 µm, and densified in the hot press using the same parameters as those mentioned for C12–SiOC.

C1–SiOC was derived from a sol–gel process established by Soraru et al. [3]. Triethoxysilane (97%, ABCr, Karlsruhe, Germany) and methyldiethoxysilane (97%, ABCr, Karlsruhe, Germany) were mixed under vigorous stirring in a molar ratio of 2:1. Polymerization was started at neutral pH with an equimolar amount of deionized water with respect to the molar amount of ethoxy groups present in the two alkoxysilanes. The sol was vigorously stirred for 15 min at ambient conditions, and subsequently filled in rectangular polystyrene boxes (50 × 50 × 190 mm^3^) with a closed lid. The lid was additionally sealed with parafilm to allow a slow and steady progress of the sol–gel reaction, as a fast reaction resulted in cracking of the monolithic pieces. The samples were aged for four weeks. The obtained monolithic xerogels had an approximate thickness of 1.15 mm. The samples were dried in temperature steps of 20 °C per day until 120 °C. One part of the samples was pyrolyzed in an alumina tube furnace at 1100 °C for three hours under flowing argon atmosphere using a slow heating rate of 25 °C/h. Faster heating resulted in cracking of the monolithic pieces. The second part of the samples was used to prepare SiOC powder for densification in a uniaxial hot press. The experimental parameters of the hot-pressing procedure were the same as for sample C12–SiOC.

*Materials characterization.* The chemical composition of the samples was determined using a carbon analyzer Leco-200 (Leco Corporation, St. Joseph, MI, USA) and a N/O analyzer Leco TC-436 (Leco Corporation, St. Joseph, Michigan, USA). The silicon weight fraction was calculated as the difference to 100 wt %, assuming that no other elements were present in the samples. Archimedean (skeletal) density and open porosity was measured with the water immersion technique. The closed porosity was measured via He pycnometry on a Pycnomatic ATC pycnometer (Porotec, Hofheim am Taunus., Germany) on finely ground powders in order to ensure full access to the total surface. Powders were ground for 2 × 60 min in a horizontal mixer mill in ZrO_2_ grinding containers. The relative density was averaged from three individual measurement cycles. Each measurement cycle consisted of several individual measurements (full evacuation and subsequent purging of the chamber). A cycle was finished when five individual values were detected within a standard deviation of 0.2%. The closed porosity was calculated from the percental deviation from the skeletal density determined by the water immersion technique. Powder XRD measurements were performed in flat-sample transmission geometry on a STOE STADI P diffractometer (Stoe, Darmstadt, Germany) equipped with a Molybdenum X-ray tube and a position sensitive detector with a 6° aperture. Raman spectra were recorded on a Horiba HR800 micro-Raman spectrometer (Horiba JobinYvon, Bensheim, Germany) equipped with a He–Ne laser (633 nm). The measurements were performed by using a grating of 600 g/mm and a confocal microscope (magnification 50 × NA 0.75–numerical aperture) with a 100-μm aperture, giving a resolution of approximately 1 μm. The laser power (20 mW) was attenuated by using neutral density filters.

*Measurement of the thermal properties.* The coefficient of thermal expansion (CTE) was measured using a dilatometer (DIL 402 E, NETZSCH Gerätebau GmbH, Selb, Germany) and an Al_2_O_3_ standard with a contact force of approximately 0.25 N. Samples prepared at 1600 °C were cut in pieces with the approximate dimension of 3 mm × 4 mm × 25 mm. Sample C1–SiOC–1100 could only be prepared in the dimensions of 0.7 mm × 4 mm × 15 mm. The measurements were performed under constant argon flow, and with heating and cooling rates of 5 K/min. The CTE was determined from the slope of the linear part of the dilatometric curves, i.e., between room temperature and 1000 °C. The specific heat capacity (*C_p_*) was determined from differential scanning calorimetry (DSC) measurements. All of the DSC experiments were realized in a STA 449 F3 Jupiter (Netzsch Gerätebau GmbH, Selb, Germany), equipped with a type-S thermocouple. DSC measurements were performed in Pt crucibles with alumina inliners from room temperature to 1000 °C under argon atmosphere with a heating rate of 20 K/min. *C_p_* values were calculated relating to sapphire samples following DIN standard 51007. The thermal diffusivity was determined using a Laser Flash LFA 1600 instrument (Linseis Messgeräte GmbH, Selb, Germany) equipped with a type-S thermocouple. The measurements were conducted in vacuum using graphite sample holders. A graphite sample was measured in parallel as reference material.

## 3. Results and Discussion

### 3.1. Chemical and Microstructural Characterization of SiOC Samples 

**Figure 1:**
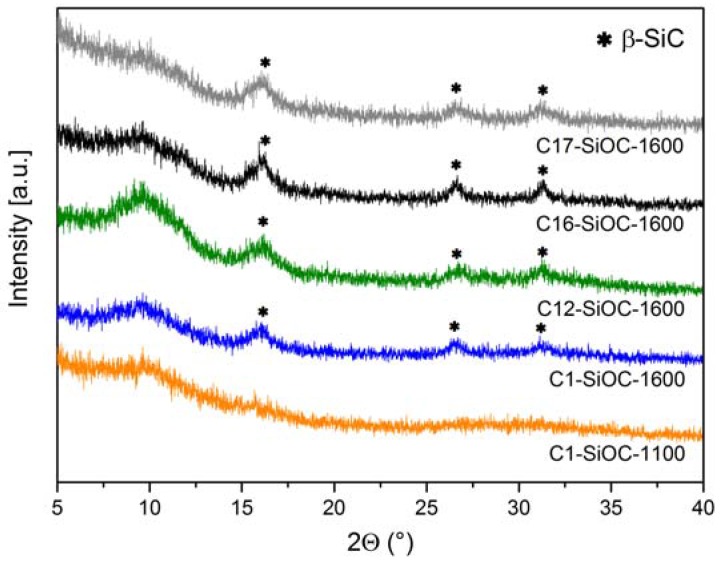
XRD patterns (λ = Mo K_α_) of the investigated silicon oxide carbides (SiOC) materials [24].

The XRD patterns of the samples investigated in this study are shown in Figure 1. Sample C1–SiOC–1100 was fully X-ray amorphous; whereas all of the other samples revealed the presence of broad reflections related to nanocrystalline β-SiC [24]. The broad hump centered at a *2θ* value of approximately 9.5° is related to the presence of amorphous silica.

**Figure 2:**
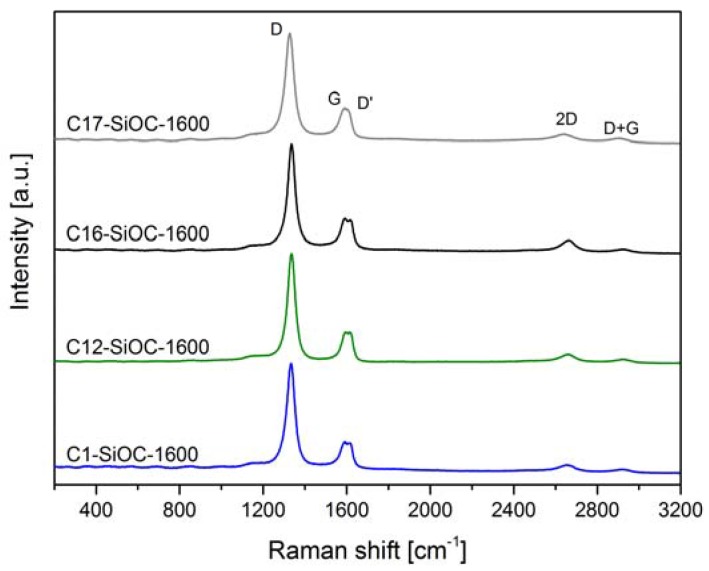
Raman spectra of the hot-pressed SiOC glass ceramics.

The chemical composition of the investigated SiOC samples is summarized in Table 1. It is evident that the phase separation has no significant influence on the chemical composition (compare sample C1–SiOC–1100 and C1–SiOC–1600). As previously proven by the ^29^Si MAS NMR spectra of samples C1–SiOC and C12–SiOC, which were prepared at 1600 °C [25,26], and the XRD patterns and Raman spectra of the studied samples shown in Figure 1 and Figure 2, the hot-pressed samples can be considered as glass ceramics consisting of an amorphous silica matrix in which nano-sized β-SiC particles and a segregated carbon phase are dispersed. On the other hand, samples prepared at 1100 °C show a different microstructure. They are fully X-ray amorphous SiOC glasses, and exhibit SiO_4−x_C_x_ mixed-bonds tetrahedra [3]. Consequently, the phase composition of SiOC glasses and glass ceramics can be estimated by considering the absence of C–O bonds in SiOC materials [1]. In the case of SiOC glasses, molar fractions of SiC and SiO_2_ can be regarded as the amount of Si–C and Si–O bonds, respectively. The segregated carbon content increased from a very limited amount in C1–SiOC–1600 (i.e., ca. 0.1 vol %) to a rather large fraction in C17–SiOC–1600 (i.e., ca. 17.4 vol %). The volume fraction of SiC was comparable in C1–SiOC–1600 and C16–SiOC–1600 (16.8 and 18.3 vol %, respectively), and only slightly lower in C12–SiOC–1600 (i.e., 12.3 vol %). Sample C17–SiOC–1600 has a significantly higher amount of SiC in comparison to the other samples, i.e., 29.1 vol %.

Raman spectroscopy is a powerful tool to investigate carbon-containing materials. The first order Raman spectrum of sp^2^ carbon exhibits a band of E_2g_ symmetry that relates to the bond stretching of sp^2^ carbon pairs that are contained in rings or chains. This band is called G band, and appears at around 1575–1595 cm^−1^. Disordered or nanostructured carbon-based materials exhibit additional bands in their first order Raman spectrum, such as: a band of A_1g_ symmetry, which relates to the breathing modes of sp^2^ carbon atoms within rings (the so-called D band; its position depends on the laser wavelength; ca. 1350 cm^−1^ at 514.5 nm); a band related to C–C sp^3^ vibrations (ca. 1150–1200 cm^−1^; can be observed upon UV laser excitation); a D'' band (ca. 1500 cm^−1^), which relates to amorphous carbon; and a D' band (ca. 1620 cm^−1^). Further important features in the Raman spectrum of disordered carbon materials are the two-dimensional (2D) band (*λ* ≈ 2500–2800 cm^−1^) and the D + G band (λ ≈ 2900 cm^−1^), representing overtone and combinational modes, respectively [27].

Besides C1–SiOC–1100, which is lacking any bands in the Raman spectrum (not shown), and is suffering from large fluorescence, all of the samples that were hot-pressed at 1600 °C showed rather similar Raman spectra, and revealed the typical features of a disordered sp^2^-hybridized segregated carbon phase, as shown in Figure 2.

This can be further illustrated by the comparison of some calculated graphitization indicators. The ratio between the D and the G bands gives information about the lateral crystal size *L_a_* of the individual domains of the segregated carbon phase, which can be estimated by using the equation given in Cançado et al. [28] (Equation (1)). Similarly, the average inter-defect distance, *L_D_*, can be calculated from Equation (2) [29].

(1) $$L_{a}\left(nm\right)=2.4\cdot 10^{-10}\cdot {\lambda_{L}}^{4}\cdot {\left(\frac{A_{D}}{A_{G}}\right)}^{-1}$$

(2) $${L_{D}}^{2}\left(nm^{2}\right)=1.8\cdot 10^{-9}\cdot {\lambda_{L}}^{4}\cdot {\left(\frac{A_{D}}{A_{G}}\right)}^{-1}$$

with *λ_L_* being the laser wavelength in nm, as well as *A_D_* and *A_G_* representing the integrated area of the D band and the G band, respectively. Larouche et al. defined the value *L_eq_*, which describes the average continuous carbon precipitate size, including tortuosity [30], and can be calculated from the ratio *A_2D_/A_D_*, as shown in Equation (3).

(3) $$L_{eq}\left(nm\right)=77.0648\cdot \left(\frac{A_{2D}}{A_{D}}\right)$$

The values for *L_a_*, *L_D_*, and *L_eq_* are listed in Table 2. No significant difference is observed for the investigated SiOC glass ceramics. Thus, it is concluded that the segregated carbon phase has a comparable degree of graphitization for all of the SiOC glass ceramics, independently of their chemical composition, as already shown by Roth et al. for SiOC samples prepared at 1600 °C with an intermediate amount of segregated carbon [31]. The average lateral crystal size is in the same range as the mean inter-defect distance. The equivalent size L_eq_ is only slightly larger than *L_a_*, indicating that the carbon precipitates are nanoscaled. Moreover, the width of the D and G bands ranges between 40 and 50 cm^−1^, which is close to the values known for glassy carbon (wD = 52.7 cm^−1^; wG = 56.1 cm^−1^) [32].

The skeletal density of SiOC increased upon phase separation, as shown in Table 3 by the comparison of the values for C1–SiOC–1100 and C1–SiOC–1600, whereas the chemical composition does not strongly affect the density of SiOC glass ceramics.

All of the monolithic samples prepared in this study, except C17–SiOC–1600, are fully dense and crack-free, as evidenced by the measurements of the open and closed porosity (*cf.* Table 3), as well as scanning electron microscopy (SEM). C17–SiOC–1600 reveals only little open porosity (i.e., 1.6 vol %), but comprises ca. 7 vol % closed porosity, as determined by helium pycnometry. Thus, apart from C17–SiOC–1600, the samples are suitable for measuring the intrinsic thermal properties of SiOC glasses and glass ceramics. For C17–SiOC–1600, values are expected to be underdetermined due to the presence of closed porosity [33].

### 3.2. Thermal Properties of SiOC Glasses and Glass Ceramics

The thermal expansion and specific heat capacity of SiOC glass and glass ceramics were assessed. Moreover, the thermal diffusivity was measured, and used to rationalize the thermal conductivity in the studied samples. The temperature-dependent thermal conductivity *λ(T)* can be calculated according to Equation (4):

(4) $$\lambda \left(T\right)=\alpha \left(T\right)\cdot C_{p}\left(T\right)\cdot \rho \left(T\right)$$

where *α(T)* is the temperature-dependent thermal diffusivity, *C_p_(T)* is the temperature-dependent specific heat capacity, and *ρ(T)* is the temperature-dependent density. At temperatures exceeding 900 °C, values for the specific heat capacity and thermal diffusivity were extrapolated (neglecting possible glass transitions). For the sample C1–SiOC–1100, the phase separation starting at approximately 1200 °C [7] was neglected upon extrapolation. In the following, the results of the multimethod approach used for the rationalization of the thermal transport in silicon oxide carbide are described and discussed.

**Figure 3:**
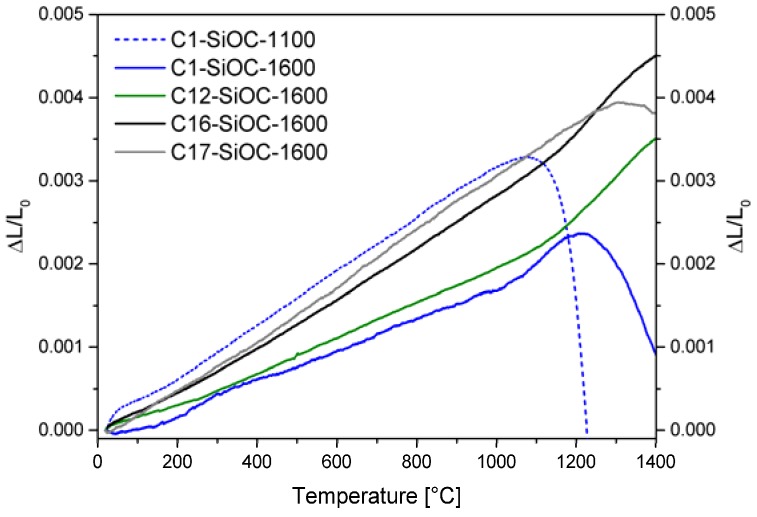
Thermal expansion of a SiOC glass and SiOC glass ceramics. The heating rate during the dilatometric experiments was 5 K/min.

The coefficient of thermal expansion (CTE) values determined for the SiOC glass ceramics between 100–1000 °C (Figure 3 and Table 4) are nearly one order of magnitude higher in comparison to that of vitreous silica (CTE = 5.7 × 10^–7^ K^–1^, [14]), but are still very low. They are close to the values reported by Renlund et al. [13] (CTE = 3.14 × 10^–6^ K^–1^) for a SiOC glass ceramic with a composition similar to that of C12–SiOC–1600. For SiOC glass ceramics, the thermal expansion increased as the carbon content increased. This is in agreement with pyrolytic carbon and β-SiC having larger CTE values of 4–6 × 10^−6^ K^−1^ [34] and 4.3–4.9 × 10^–6^ K^–1^ [35], respectively. The thermal expansion of C1–SiOC–1100 was already investigated by Rouxel et al. [14], and determined to be 3.12 × 10^–6^ K^–1^. This is very close to the value of 3.23 × 10^–6^ K^–1^ determined in this study, and consequently higher in comparison to that of C1–SiOC–1600. Thus, the phase separation of silicon oxide carbides leads to a decrease in their thermal expansion, following the lower value for vitreous silica. Upon the incorporation of additional carbon, the CTE of the silicon oxide carbide glass ceramics can be raised again to the values of the SiOC glass (*cf*. C1–SiOC–1100 vs. C17–SiOC–1600, both with a CTE value of 3.23 × 10^–6^ K^–1^). This proves the possibility of adjusting/tailoring the thermal properties of SiOC materials by altering the content of their segregated carbon phase.

At higher temperatures, samples C1–SiOC–1600, C12–SiOC–1600, and C16–SiOC–1600 reveal an increase in the values for CTE (*cf.* CTE_HT_ in Table 4). This behavior is also known in the literature for other glass systems [36,37]. The values for CTE_HT_ of SiOC glass ceramics are higher than the CTE values at 100–1000 °C by a factor of ca. 2. This increase in the CTE values has to be considered when anticipating applications of SiOC materials at temperatures beyond 1000 °C.

For the calculation of the temperature-dependent thermal conductivity, knowledge of the temperature-dependent densities of the SiOC materials is necessary. These can be calculated from the CTEs under the assumption of isotropic expansion, as expected for amorphous materials. Furthermore, it has to be noted that the influence of the temperature on the density of SiOC is small due to the low thermal expansion.

All of the SiOC glass ceramics except C17–SiOC–1600 showed a monotonic expansion, followed by an increase in the slope at higher temperatures. From this kink, the glass transition temperature *T_g_* can be estimated by the intersection of the linear slopes to the left and right of the kink. They were determined to be 1060 °C for C1–SiOC–1600, 1157 °C for C12–SiOC–1600, and 1171 °C for C16–SiOC–1600. The values for *T_g_* were approximately 140–170 °C lower than those derived from the temperature dependence of the shear viscosity obtained via compression creep measurements (data not shown), and even lower than those reported for vitreous silica (i.e., ~1190 °C depending on the amount of impurities [38]). Within this context, it should be mentioned that in various studies, the impact of the applied pressure in the dilatometer on the determined *T_g_* value has been considered to be significant, and thus, a high mechanical load during the dilatometry experiments was shown to lead to relatively low *T_g_* values (as with respect to values determined by other methods), while the values of the CTE remain unbiased [39]. As the focus of the present study was the accurate determination of the thermal expansion, the specimens were thoroughly fixed in the dilatometer (load of 0.25 N, corresponding to 0.02 MPa). Consequently, the determined *T_g_* values were considered as being altered by the mechanical load used, and were thus less accurate than those determined from creep experiments [25,26,40].

An additional parameter that can be determined from the thermal expansion curve is the dilatometric softening point corresponding to the maximum of the dilatometric curve, and to a viscosity of ca. 10^10^ Pa∙s. Among the SiOC glass ceramics studied, such a maximum is visible for samples C1–SiOC–1600 at 1220 °C, and for C17–SiOC–1600 at 1300 °C, which is again significantly lower than those derived from the shear viscosities. Similar to *T_g_*, the dilatometric softening point was dependent on the applied pressure for fixing the samples inside the dilatometer [39]. With increasing pressure, the dilatometric softening point was shifted to lower temperatures. Moreover, the value determined for sample C17–SiOC–1600 was considered as being biased by a slight densification due the elimination of the residual porosity.

C1–SiOC–1100 showed a monotonic increase in the thermal expansion up to 1070 °C. At higher temperatures, a rapid shrinkage was observed, as already described by Rouxel et al. [14] for the same SiOC glass composition. The C1–SiOC–1100 specimen investigated in this study was slightly bent after the measurement, most probably due to viscous flow. However, the measured specimen was very thin (0.7 mm), and the softening point was considered as being biased, as Euler’s critical load is dependent on the second moment of area [41]. Consequently, neither the glass transition temperature nor the softening point could be evaluated for sample C1–SiOC–1100.

**Figure 4:**
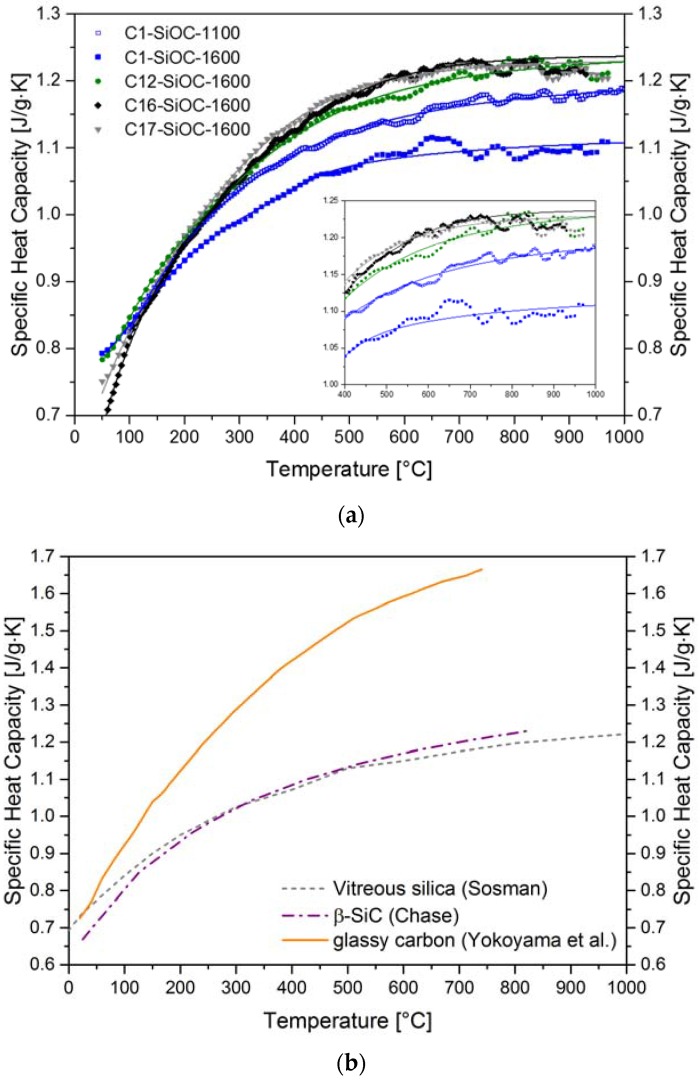
Temperature-dependent specific heat capacities of SiOC glasses and glass ceramics (**a**). Points represent the measured values; the solid lines represent eye guidelines. The inset details the specific heat capacities in the temperature range of 400 °C to 1000 °C. The values for glassy carbon [44], vitreous silica [42], and β-SiC [43] are plotted for comparison (**b**).

The evolution of the specific heat capacity with the increasing temperature of the SiOC materials and glassy carbon is shown in Figure 4. The values were in the range as expected for ceramics, and rather similar to those known for vitreous silica [42] and β-SiC [43]. The temperature dependence of the specific heat capacity of samples with low contents of carbidic carbon (C1–SiOC and C12–SiOC) resembled that of vitreous silica, whereas that of the samples with a higher carbidic carbon content (C16–SiOC and C17–SiOC) resembled that of β-SiC, revealing a steeper slope in their linear range. C1–SiOC–1100 showed a nearly identical behavior to vitreous silica, up to ca. 700 °C. The phase separation lead to a decrease in the specific heat capacity, probably due to the increase in density during this process (*cf.* Table 3), as evidenced by the comparison of C1–SiOC–1100 and C1–SiOC–1600. On the other hand, an increase in the specific heat capacity could be observed for C12–SiOC–1600, C16–SiOC–1600, and C17–SiOC–1600, with no significant differences at 1000 °C. The similar molar masses and densities of the three investigated SiOC glass ceramics was considered to be the main reason for the nearly identical specific heat capacities at 1000 °C.

**Figure 5:**
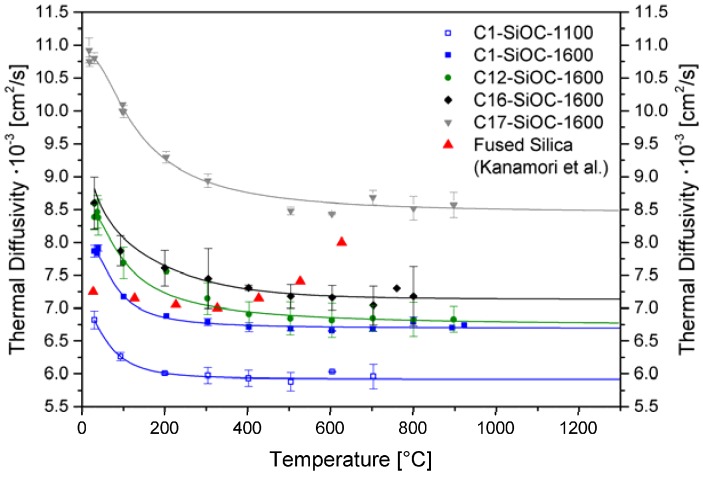
Thermal diffusivity of SiOC glass and glass ceramics. The data points represent the values measured, while the solid lines represent guidelines for the eyes. The data for fused silica are taken from Kanamori et al. [45].

Figure 5 summarizes the thermal diffusivity of the investigated SiOC glass and glass ceramics. The low carbon-containing SiOC glass ceramics revealed comparable values to fused silica [45]. The thermal diffusivity in SiOC increased as the amounts of segregated carbon increased. This observation matched the higher thermal diffusivity values for glassy carbon of 0.05 cm^2^/s to 0.044 cm^2^/s in the temperature range between 20–700 °C [44]. However, the amount of carbidic carbon (i.e., volume fraction of β-SiC) additionally seemed to have an important influence. This is expressed by the comparison of C16–SiOC–1600 and C17–SiOC–1600, as their amount of segregated carbon was comparable; however, their thermal diffusivities were significantly different. It is conceivable that the comparably high values of C17–SiOC–1600 were resulting from the higher amount of β-SiC (*cf.* Table 1), which showed significantly higher thermal diffusivity values of 0.809 cm^2^/s at room temperature [46].

Interestingly, sample C1–SiOC–1100 showed lower values for the thermal diffusivity in comparison to the phase-separated SiOC glass ceramics, as well as to fused silica (see Figure 5). Two aspects are considered here: (i) SiOC glasses prepared at 1000–1100 °C are known to possess a significant amount of hydrogen [10], and most probably a significant content of dangling bonds. These are expected to act as phonon scatterers; (ii) the unique network architecture of the SiOC glass, which was shown to be characterized by a low mass fractal dimension (~2.5) [1], was also considered to be an important reason for the reduced thermal diffusivity in SiOC glass. It was shown in numerous papers that fractal networks (such as silicate-based or vitreous silica) show anomalous behavior with respect to heat transport, and that this is correlated to their fractal architecture [47,48,49]. However, it is still not clear whether and to which extent the mass fractal dimension of a network alters its thermal transport. Thus, additional and more detailed theoretical and experimental investigations are needed for our silicon oxide carbide glasses and glass ceramics in order to elucidate this aspect.

Based on the thermal diffusivity data determined for glassy SiOC (C1–SiOC–1100), as well as the comparison with its phase-separated analogous material (C1–SiOC–1600), it can be concluded that the phase separation in SiOC leads to higher values of thermal diffusivity. This is an obvious effect, if we assume that the network architecture of the silica matrix in SiOC glass ceramics is comparable to vitreous silica. Furthermore, at a synthesis temperature of 1600 °C, significantly lower values of hydrogen in the resulting SiOC glass ceramics were present, as evidenced by Soraru et al. and Brequel et al. [3,10].

**Figure 6:**
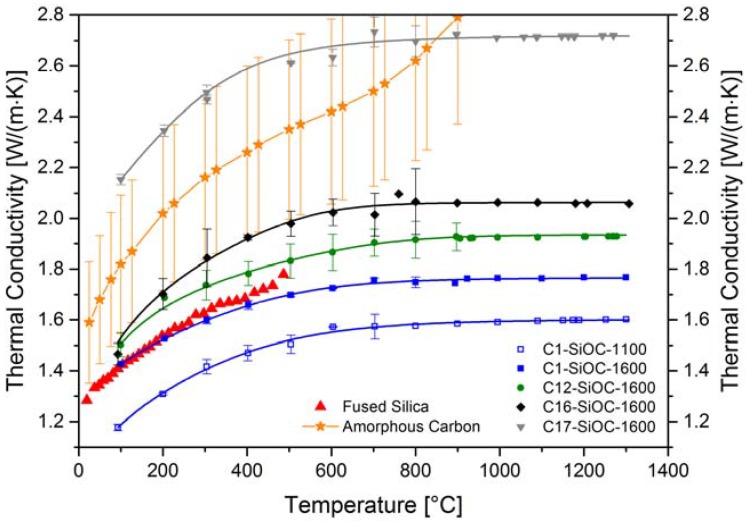
Thermal conductivity of SiOC glasses and glass ceramics. The data points represent the values measured; the solid lines are a guide for the eyes. The standard deviation is taken as the relative standard deviation according to the thermal diffusivity measurements. Data for fused silica and for amorphous carbon are taken from Cahill [52] and Ho et al. [53], respectively.

The thermal conductivity was calculated according to Equation (1) using the thermal properties presented above. Figure 6 summarizes the thermal conductivities obtained for the SiOC glass and glass ceramics. The values for C17–SiOC–1600 were corrected with respect to their open and closed porosity, as porosity reduced the thermal transport [33]. The impact of the open porosity was corrected in a first step. For materials with small fractions of open porosity (<10 vol %), the relation developed by Loeb [50] (*cf.* Equation (5)) was reported to yield appropriate values for their effective thermal conductivity [33]. As a second step, the impact of the closed porosity was corrected using the Maxwell–Eucken equation [51] (*cf.* Equation (6)). This equation was reported to appropriately taking into account fractions of < 15 vol % of closed porosity that are homogeneously dispersed in a solid matrix [33].

(5) $$\lambda_{eff}=\lambda_{s}\left(1-v_{p}\right)$$

(6) $$\lambda_{eff}=\lambda_{s}\frac{\lambda_{p}+2\lambda_{s}+2v_{p}\left(\lambda_{p}-\lambda_{s}\right)}{\lambda_{p}+2\lambda_{s}-v_{p}\left(\lambda_{p}-\lambda_{s}\right)}$$

In the above equations, *λ_eff_* is the effective thermal conductivity of the porous sample, *λ_s_* is the thermal conductivity of the solid pore-free sample, *λ_p_* is the thermal conductivity through the pores, and *v_p_* is the volume fraction of the pores. *λ_p_* is expected to be negligible.

Among all of the studied samples, the glassy SiOC material (C1–SiOC–1100) showed the lowest thermal conductivity, whereas the phase-separated low carbon-containing SiOC glass ceramic sample (C1–SiOC–1600) showed values very similar to vitreous silica [52] (Figure 6). As the content of segregated carbon increased in the SiOC glass ceramics, the thermal conductivity was observed to increase. This is probably due to the higher thermal conductivity value of the segregated carbon present in the materials. For instance, amorphous carbon [53] exhibited values in the range of the high carbon-containing SiOC glass ceramics up to 800 °C (C16–SiOC–1600 and C17–SiOC–1600), and reached values of ca. 3.18 W/(m∙K) at 1000 °C. Interestingly, the thermal conductivity of C17–SiOC–1600 was significantly higher than that of C16–SiOC–1600, despite there being a comparable content of segregated carbon present in both samples (i.e., 16 vs. 17 vol %). The reason for this remarkable difference is considered to rely on the different content of the nanoscaled silicon carbide phase present in the mentioned samples. As crystalline β-SiC exhibited comparatively large thermal conductivities of 178.2 W/(m∙K) [46], the increased volume fraction of silicon carbide in C17–SiOC–1600 may indeed be the reason for its higher thermal conductivity, as compared to C16–SiOC–1600.

The only available data on the temperature-dependent thermal transport in SiOC-based materials in the literature has been shown and discussed in Gurlo et al. [21]. It was stated that the thermal transport in multiphasic SiOC materials (i.e., glassy SiOC with additional segregated carbon or SiOC glass ceramics containing segregated carbon and additional disperse phases such as silicon carbide) should be governed by the percolating phases [21] present in their microstructures. In the case study mentioned [21], and for our sample C1–SiOC–1600, the only percolating phase was the glassy silica matrix. Sample C12–SiOC was close to the percolation threshold of segregated carbon, as recently evidenced by Roth et al. via electrical conductivity measurements [31]. For C16–SiOC–1600 and C17–SiOC–1600, a percolating path of the segregated carbon phase was expected to be present due to its higher volume fraction. Indeed, samples C12–SiOC–1600 to C17–SiOC–1600 exhibited an increase of the thermal conductivity as the segregated carbon content increased, getting closer to the typical values for amorphous carbon. However, this increase did not scale linearly with the amount of segregated carbon. A varying degree of ordering (i.e., graphitization) of the segregated carbon phase in the different SiOC glass ceramics can be ruled out, as evidenced by the Raman spectroscopy data presented above (Table 2). Sample C17–SiOC–1600 revealed a significantly higher thermal conductivity, which was most probably due to the higher content of β-SiC nanoparticles, as discussed already for the thermal diffusivity. Consequently, a simple Maxwell–Garnett model [54,55] for the description of its thermal conductivity, where a thermal interaction of dispersed particles is ignored, may be not suitable for sample C17–SiOC–1600.

There are several models proposed in the literature for the calculation of the effective thermal conductivity of composite materials at different border conditions. As already pointed out, a percolating phase is expected to behave differently than an isolated phase, and for our samples, both situations have to be considered. Kingery pointed out that for a two-phase system, the effective thermal conductivity depends on the conductivity of both phases (regardless whether they are continuous or not), and on their distribution [56]. However, a continuous phase dominates the effective thermal conductivity of the composite [56]. For composites with more than one continuous phase, i.e., interpenetrating phase composites (IPC), the description of the effective thermal conductivity is more complex. However, upon the comparison of a model developed for liquid-phase sintered Cu/W IPCs, a linear dependence of the effective thermal conductivity on the volume fractions of the interpenetrating phases may be expected [57]. This model seems to fit our samples well, as in the model, copper as the high conducting phase (and in our samples, the segregated carbon phase) has lower volume fractions than the lower conducting phase tungsten (in our samples, glassy SiO_2_). This is especially important, as the different models for the effective thermal conductivity of composites yield different results depending on which phase (isolated, continuous, etc.) is the higher or lower conducting phase [58].

**Figure 7:**
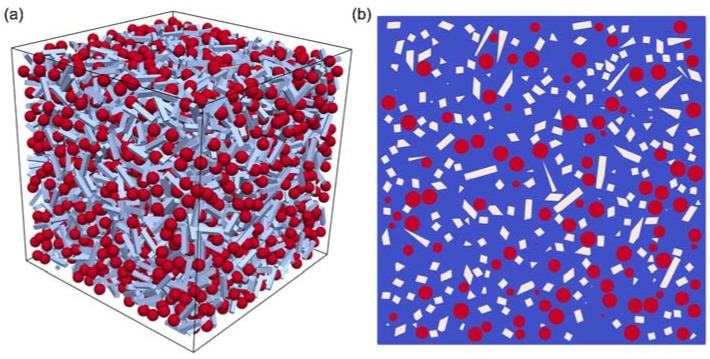
Modeled microstructure of sample C12–SiOC–1600: (**a**) three-dimensional representation of a 100 × 100 × 100 nm^3^ volume and (**b**) two-dimensional cross-section of (**a**). Red spheres represent β-SiC nanoparticles (5 nm diameter), and white rods represent the segregated carbon phase.

Sample C1–SiOC–1600 consisted of ca. 83 vol % glassy SiO_2_ and ca. 17 vol % dispersed particles (β-SiC). The continuous phase was the glassy SiO_2_ matrix. For sample C12–SiOC–1600, the glassy SiO_2_ matrix was forming a continuous phase, and the segregated carbon was expected to form at least partially percolating paths. The effective thermal conductivities of both of these material mixtures (C1–SiOC–1600, C12–SiOC–1600) have been computed by means of a first-order computational homogenization scheme. The SiC inclusions and the segregated carbon phase have been approximated by spherical particles with a diameter of 5 nm, and by rods with dimensions of 10 × 2 × 2 nm^3^, respectively. These particles were randomly placed in a sample volume of fused silica with dimensions 100 × 100 × 100 nm^3^. Insertion continued until reaching the desired volume fractions of 12.3 vol % for SiC, and 11.9 vol % for carbon for C12–SiOC–1600. Figure 7a shows a view of the particle distribution in sample C12–SiOC–1600. A cross-section of sample C12–SiOC–1600 is given in Figure 7b, and exhibits good agreement with the sample microstructure as observed by TEM [26,40], particularly with respect to the interparticle spacing.

**Figure 8:**
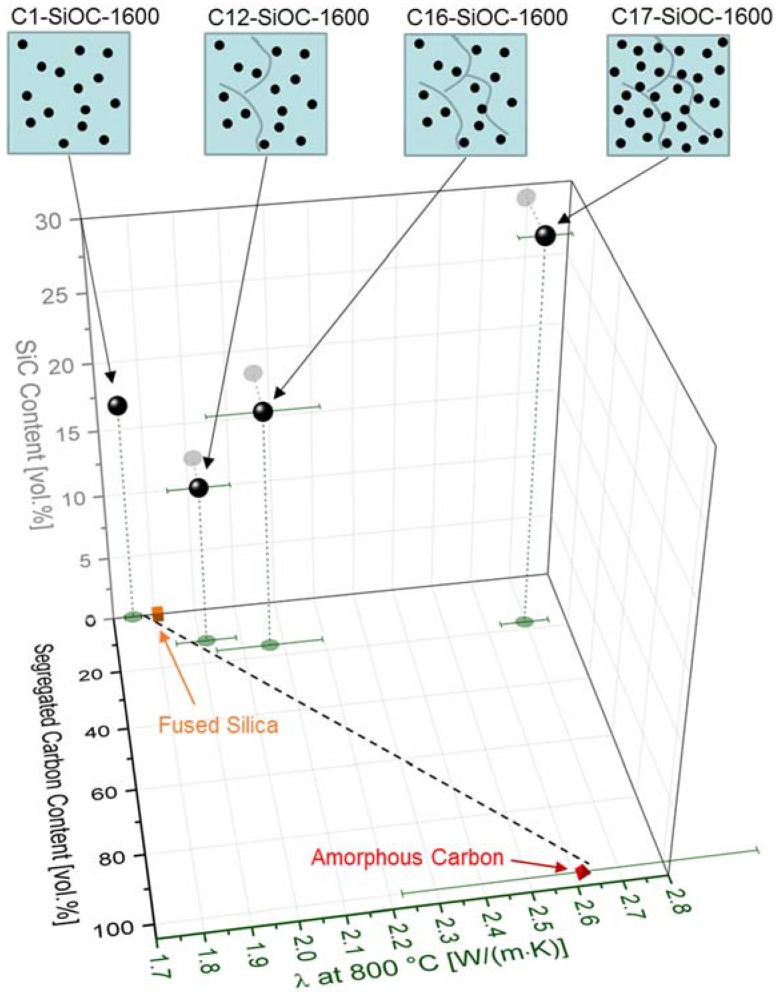
Thermal conductivity *λ* of the SiOC glass ceramics investigated in this work, and of reference materials at 800 °C, as a function of the segregated carbon content and the SiC content. Data for fused silica was extrapolated from Cahill [52], and data for amorphous carbon was taken from Ho et al. [53]. Two-dimensional (2D) projections of the individual values on the xy plane and on the yz plane are included as green and grey transparent dots, respectively. Sample C1–SiOC–1600 and sample C12–SiOC–1600 can be effectively described as a linear mixture between the two percolating phases (*cf*. trend line), namely, the glassy SiO_2_ matrix and the segregated carbon phase. The β-SiC particles possibly show a slight impact for C16–SiOC–1600, and a significant impact for C17–SiOC–1600. They are expected to have a strong contribution to the thermal transport as part of a high-λ percolating phase consisting of segregated carbon and β-SiC. The highly schematic images of the respective microstructures are indicated for SiOC glass ceramics at the top insets, in which the black dots represent β-SiC, and the grey lines represent the segregated carbon phase.

The effective thermal conductivity tensor was determined from the solution of a Laplace problem with three sets of linear temperature boundary conditions, each with respect to one of the spatial directions. The employed thermal conductivities for fused silica, SiC, and segregated carbon read 1.75 W/(m∙K) [52], 5 W/(m∙K) [59,60], and 2.5 W/(m∙K) [53], respectively. The trace of the conductivity tensor yielded the desired isotropic thermal conductivity of the material mixture. For sample C12–SiOC–1600, the thermal conductivity was found to exceed that of fused silica by a factor of 1.22, possessing a value of approximately 2.14 W/(m∙K). This is significantly higher than the experimental determined values. However, the numerical simulations were performed upon neglecting the thermal contact resistance at the interface between the particles and the matrix. We assume that this was due to the high total surface area of the nano-sized β-SiC particles, which summed up to a significant total thermal contact resistance between the β-SiC particles and the matrix. As a consequence, the values of the thermal conductivity were lowered. If the segregated carbon phase was present as a percolating phase, its thermal conductivity had a linear impact on the effective thermal conductivity relative to its volume fraction [57]. This concept is depicted in Figure 8 for thermal conductivity values at 800 °C as a function of the volumetric amount of segregated carbon phase and β-SiC. A linear trend line was drawn between the first percolating phase (fused silica) and the second percolating phase (amorphous carbon). Samples C1–SiOC–1600 and C12–SiOC–1600 both fell on this line, supporting the linear relation between the interpenetrating phases.

C16–SiOC–1600 fell slightly off the linear trend line. However, a clear statement is difficult, as the rather high standard deviation of the thermal conductivity of amorphous carbon hampered the conclusions. Most probably, C16–SiOC–1600 had two percolating phases, namely, the glassy SiO_2_ matrix, and the segregated carbon phase. A contribution of the β-SiC nanoparticles to the percolating carbon phase cannot be excluded. However, as the thermal conductivity of C17–SiOC–1600 is by far higher than that expected from a linear mixture between the two percolating phases (glassy SiO_2_ matrix and segregated carbon phase), a contribution of the SiC nanoparticles is most probable. As already discussed for the thermal diffusivity, the β-SiC particles are expected to be the reason for this steep increase, as C17–SiOC–1600 possessed almost 30 vol % β-SiC nanoparticles. Consequently, for C17–SiOC–1600, two percolating phases were expected to be present, namely the low thermal conductivity (low-λ) glassy SiO_2_ matrix, and a high-λ percolating path consisting of β-SiC and segregated carbon (dominated by the contribution of SiC).

Gurlo et al. [21] investigated a hot-pressed material, which was synthesized from the same polymer as sample C12–SiOC–1600 in this study, and additionally, the same chemical composition prepared at 1100 °C. It is important to note that the latter sample was obtained from a self-filler process, and the final monolith showed a considerable porosity of 12 vol %. Additionally, the self-filler technique introduces additional grain boundaries, and consequently increases the overall interfacial thermal resistance. The sample with the composition equivalent to C12–SiOC–1600 showed slightly lower thermal conductivities, comparable to those reported here for C1–SiOC–1600. The sample prepared at 1100 °C possessed considerably lower values of about 0.5 to 0.7 W/(m∙K). Upon comparison with samples C1–SiOC–1100 and C1–SiOC–1600 in this study, it can be concluded that the low values from Gurlo et al. [21] are probably related to the porosity, as indicated in the study, as well as additional grain boundaries originating from the self-filler-assisted synthesis [21].

The thermal conductivity of sample C1–SiOC–1100 from our study can be considered as being representative of the intrinsic thermal transport of the glass matrix in SiOC glasses, as it possesses only a very limited amount of segregated carbon and no porosity.

## 4. Conclusions

In the present study, the effect of the chemical/phase composition and the microstructure of SiOC materials on their thermal properties was investigated. Values of the heat capacity, thermal diffusivity, and thermal conductivity of glassy SiOC with no segregated carbon are similar to those reported for vitreous silica, whereas the thermal expansion in SiOC was slightly larger than that of silica. It is shown that the phase separation of SiOC glass leads to a rather significant decrease of the coefficient of thermal expansion (i.e., from 3.2 × 10^−6^ K^−1^ in the glass to 1.8 × 10^−6^ K^−1^ in the phase-separated state), and a slight increase of the thermal conductivity (i.e., from 1.5 to 1.7 W/(m∙K)). The changes in thermal expansion and thermal conductivity, which were associated with the phase separation of the SiOC glass network, could be adjusted by tuning the content of segregated carbon (which may be present in the glassy, as well as the phase-separated state), and of SiC nanoparticles, which were in situ generated upon the phase separation of the SiOC glass network. Thus, fractions of ca. 10 vol % of segregated carbon are sufficient to modify the thermal expansion of phase-separated SiOC from 1.8 × 10^−6^ K^−1^ back to its value from the single-phase glassy state. Increasing the content of segregated carbon (i.e., up to 12–15 vol %) and SiC nanoparticles (up to 29 vol %) in the evaluated phase-separated SiOC glass ceramics lead to thermal conductivity values in the rage from 1.18 and 2.7 W/(m∙K). It is considered that tailoring the chemical/phase composition in silicon oxide carbides via suitable polymeric precursors and processing parameters enabled tuning their thermal properties.

## Figures and Tables

**Table 1:** Empirical formulae and phase compositions of the silicon oxide carbide (SiOC) samples used in this study [1].

Sample	Composition	SiO_2_ (mol %)	SiC (mol %)	C_free_ (mol %)	SiO_2_ (vol %)	SiC (vol %)	C_free_ (vol %)
C1–SiOC–1100	SiO_1.38_C_0.32_	68.1 ^1^ ± 1.1	30.7 ^1^ ± 2.3	1.2 ^1^ ± 2.9	-	-	-
C1–SiOC–1600	SiO_1.41_C_0.30_	70.2 ± 0.5	29.3 ± 2.1	0.5 ± 2.7	83.1 ± 0.4	16.8 ± 1.2	0.1 ± 0.7
C12–SiOC–1600	SiO_1.50_C_0.71_	51.3 ± 0.3	17.2 ± 1.4	31.5 ± 1.7	75.7 ± 0.4	12.3 ± 1.0	11.9 ± 0.7
C16–SiOC–1600	SiO_1.27_C_0.97_	39.6 ± 1.1	22.8 ± 1.7	37.6 ± 1.7	65.7 ± 1.9	18.3 ± 1.3	16.0 ± 0.7
C17–SiOC–1600	SiO_0.94_C_1.13_	29.5 ± 0.5	33.1 ± 1.4	37.4 ± 1.5	53.5 ± 0.9	29.1 ± 1.2	17.4 ± 0.7

**^1^** Molar fractions of silicon carbides (SiC) and SiO_2_ can be regarded as the amount of Si–C and Si–O bonds, respectively.

**Table 2:** Indicators for the degree of graphitization of the segregated carbon phase in the prepared SiOC glass ceramics.

Sample	C_segregated_ (vol %)	*A_D_/A_G_*	*L_a_* (nm)	*L_d_* (nm)	*L_eq_* (nm)
C1–SiOC–1600	0.1 ± 0.7	4.868 ± 1.074	7.9 ± 1.7	7.7 ± 0.8	10.0 ± 0.5
C12–SiOC–1600	11.9 ± 0.8	4.215 ± 0.251	9.2 ± 0.6	8.3 ± 0.3	11.0 ± 0.7
C16–SiOC–1600	16.0 ± 0.7	4.121 ± 0.521	9.5 ± 1.3	8.4 ± 0.6	11.6 ± 3.3
C17–SiOC–1600	17.4 ± 0.7	4.998 ± 0.340	7.5 ± 0.5	7.5 ± 0.2	10.1 ± 0.6

**Table 3:** Skeletal density and volume fractions of porosity in the SiOC samples investigated in this study. The density of silica is taken from Renlund et al. [13] and indicated for the sake of comparison.

Sample	Composition	Skeletal Density (g/cm^3^)	Open Porosity (vol %)	Closed Porosity (vol %)
Vitreous Silica	SiO_2_	2.20	-	-
C1–SiOC–1100	SiO_1.38_C_0.32_	2.28	0.3	-
C1–SiOC–1600	SiO_1.41_C_0.30_	2.38	0	-
C12–SiOC–1600	SiO_1.50_C_0.71_	2.31	0	-
C16–SiOC–1600	SiO_1.27_C_0.97_	2.34	0	-
C17–SiOC–1600	SiO_0.94_C_1.13_	2.33	1.6	7.0

**Table 4:** Coefficient of thermal expansion (CTE) values of SiOC glass and glass ceramics in the temperature range between 100–1000 °C. CTE_HT_ represents the thermal expansion coefficient between *T_g_* and 1300 °C.

Sample	Composition	CTE (10^−6^ K^−1^)	CTE_HT_ (10^−^^6^ K^−1^)
C1–SiOC–1100	SiO_1.38_C_0.32_	3.23	-
C1–SiOC–1600	SiO_1.41_C_0.30_	1.84	4.41
C12–SiOC–1600	SiO_1.50_C_0.71_	2.02	4.87
C16–SiOC–1600	SiO_1.27_C_0.97_	3.09	5.29
C17–SiOC–1600	SiO_0.94_C_1.13_	3.23	-
